# 561. Reduction of RSV-associated hospitalization rates during the 2024-2025 RSV Season among infants <6 months of age

**DOI:** 10.1093/ofid/ofaf695.170

**Published:** 2026-01-11

**Authors:** Ayzsa Tannis, Leah Goldstein, Geoffrey A Weinberg, Mary A Staat, Daniel C Payne, Natasha B Halasa, Leila C Sahni, Julie A Boom, Peter G Szilagyi, Janet A Englund, Eileen J Klein, Ariana Toepfer, Abigail L Salthouse, Casey M Kalman, Laura S Stewart, John Williams, Marian G Michaels, Jennifer E Schuster, Rangaraj Selvarangan, Fatimah S Dawood, Heidi L Moline

**Affiliations:** Centers for Disease Control and Prevention, Atlanta, Georgia; Centers for Disease Control and Prevention, Atlanta, Georgia; University of Rochester Sch Med & Dent, Rochester, New York; Cincinnati Children's Hospital Medical Center, Park Hills, Kentucky; Cincinnati Children's Hospital Medical Center, Park Hills, Kentucky; Vanderbilt University Medical Center, Nashville, TN; Baylor College of Medicine and Texas Children's Hospital, Houston, Texas; Baylor College of Medicine, Houston, Texas; UCLA, Los Angeles, California; Seattle Children’s Hospital/Univ. Washington, Seattle, Washington; Seattle Children's Hospital and University of Washington School of Medicine, Seatte, Washington; Centers for Disease Control and Prevention, Atlanta, Georgia; Centers for Disease Control and Prevention, Atlanta, Georgia; Centers for Disease Control and Prevention, Atlanta, Georgia; Vanderbilt University School of Medicine, Nashville, Tennessee; University of Wisconsin, Madison, Wisconsin; University of Pittsburgh/ CHP, Pittsburgh, Pennsylvania; Children's Mercy Kansas City, Kansas City, MO; Children’s Mercy Hospital, Kansas City, Missouri; CDC, Atlanta, Georgia; US-CDC, Atlanta, Georgia

## Abstract

**Background:**

Respiratory syncytial virus (RSV) is the leading cause of hospitalization among US infants. The 2024-2025 season was the first full season with widely available RSV prevention products, (nirsevimab and maternal RSV vaccination) recommended for all infants aged < 8 months. To assess the impact of these products on RSV-associated hospitalizations, we compared RSV-associated hospitalization rates in infants aged < 6 months from 2017-2020 to 2024-2025.

**Table 1.**
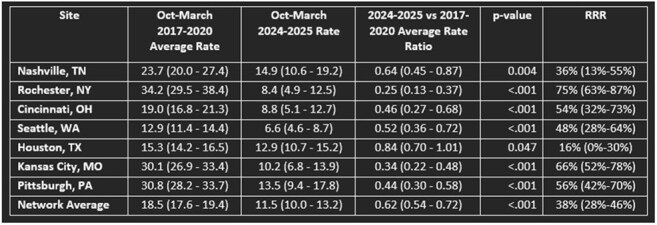
Incidence rates of RSV-associated hospitalizations per 1,000 infants by site among infants <6 months of age, New Vaccine Surveillance Network, October-March 2017-2020 and 2024-2025 (N=1,299)

**Figure 1.**
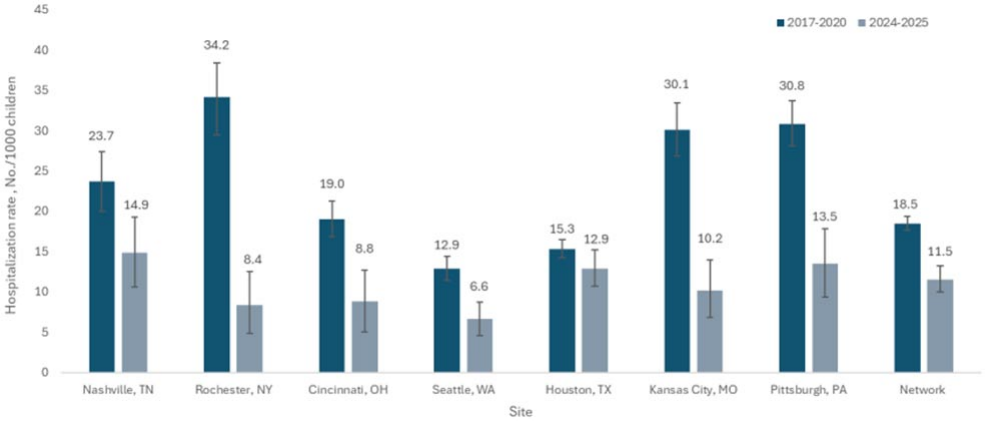
Incidence rates of RSV-associated hospitalizations per 1,000 infants by site among infants <6 months of age, New Vaccine Surveillance Network, October-March 2017-2020 and 2024-2025 (N=1,299)

**Methods:**

Infants < 6 months of age hospitalized with acute respiratory illness (ARI) were systematically enrolled at 7 US medical centers. Nasal swabs were tested for RSV by reverse transcription polymerase chain reaction. RSV circulation was determined by the percent of RSV positives among all enrolled children from September-March 2024-2025. RSV-associated hospitalizations per 1,000 infants were calculated by site during October-March 2017-2020 and 2024-2025 using US census population denominators. 95% confidence intervals (CI) were estimated by bootstrap percentiles based on 1,000 bootstrap samples. Rates were adjusted for enrollment rates, surveillance days per week, test sensitivity, and hospital market share. The relative rate reduction (RRR) was calculated as (1 – rate ratio)*100%.

**Figure 2.**
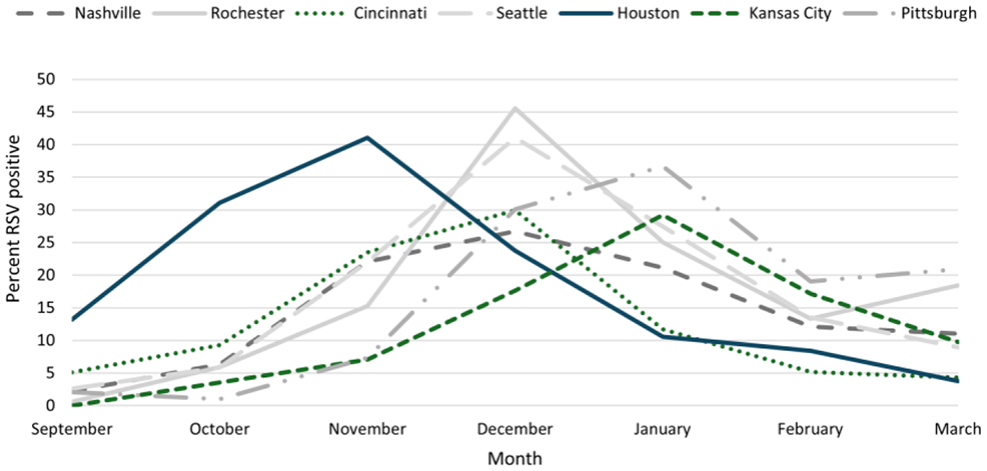
Respiratory syncytial virus (RSV) circulation by site, New Vaccine Surveillance Network, September-March 2024-2025

**Results:**

Among 1,299 infants aged < 6 months, RSV-associated hospitalization rates decreased from 18.5 (95% CI 17.6 - 19.4) in 2017-2020 to 11.5 (95% CI 10.0 - 13.2) in 2024-2025, a 38% reduction (RR 0.62, 95% CI 0.54 - 0.72). The magnitude of reduction varied by site, ranging from 16% in Houston, TX (RR 0.84, 95% CI 0.70 - 1.01) to 75% in Rochester, NY (RR 0.25, 95% CI 0.13 - 0.37). Kansas City, MO (RR 0.34 95% CI 0.22 - 0.48), Pittsburgh, PA (RR 0.44, 95% CI 0.30 - 0.58), and Cincinnati, OH (RR 0.46, 95% CI 0.27 - 0.68) also had greater than 50% reductions. RSV circulation varied, beginning in September in Houston, TX and October/November in all other sites.

**Conclusion:**

RSV-associated hospitalizations decreased 38% among infants < 6 months of age, with reductions varying by location. The magnitude of impact was reduced in sites with earlier RSV season onset, highlighting the importance of timing local product uptake relative to the RSV season in optimizing reduction of RSV-associated hospitalizations across the US.

**Disclosures:**

All Authors: No reported disclosures

